# Endometrial stromal beta-catenin is required for steroid-dependent mesenchymal-epithelial cross talk and decidualization

**DOI:** 10.1186/1477-7827-10-75

**Published:** 2012-09-07

**Authors:** Ling Zhang, Amanda L Patterson, Lihua Zhang, Jose M Teixeira, James K Pru

**Affiliations:** 1 Vincent Center for Reproductive Biology, Vincent Obstetrics and Gynecology Service, Massachusetts General Hospital/Harvard Medical School, Boston, Massachusetts, 02114, USA; 2 Department of Animal Sciences, Center for Reproductive Biology, Washington State University, Pullman, WA, 99164, USA

**Keywords:** Beta-catenin, Decidualization, Endometrium, Implantation, Pregnancy, Progesterone, Uterus

## Abstract

**Background:**

Beta-catenin is part of a protein complex associated with adherens junctions. When allowed to accumulate to sufficient levels in its dephosphorylated form, beta-catenin serves as a transcriptional co-activator associated with a number of signaling pathways, including steroid hormone signaling pathways.

**Methods:**

To investigate the role of beta-catenin in progesterone (P_4_) signaling and female reproductive physiology, conditional ablation of *Ctnnb1* from the endometrial mesenchymal (*i.e.* stromal and myometrial), but not epithelial, compartment was accomplished using the *Amhr2-Cre* mice. Experiments were conducted to assess the ability of mutant female mice to undergo pregnancy and pseudopregnancy by or through oil-induced decidualization. The ability of uteri from mutant female mice to respond to estrogen (E_2_) and P_4_ was also determined.

**Results:**

Conditional deletion of *Ctnnb1* from the mesenchymal compartment of the uterus resulted in infertility stemming, in part, from complete failure of the uterus to decidualize. E_2_-stimulated epithelial cell mitosis and edematization were not altered in mutant uteri indicating that the mesenchyme is capable of responding to E_2_. However, exposure of ovariectomized mutant female mice to a combined E_2_ and P_4_ hormone regimen consistent with early pregnancy revealed that mesenchymal beta-catenin is essential for indirectly opposing E_2_-induced epithelial proliferation by P_4_ and in some mice resulted in development of endometrial metaplasia. Lastly, beta-catenin is also required for the induced expression of genes that are known to play a fundamental role in decidualization such as *Ihh*, *Ptch1*, *Gli1* and *Muc1*

**Conclusions:**

Three salient points derive from these studies. First, the findings demonstrate a mechanistic linkage between the P_4_ and beta-catenin signaling pathways. Second, they highlight an under appreciated role for the mesenchymal compartment in indirectly mediating P_4_ signaling to the epithelium, a process that intimately involves mesenchymal beta-catenin. Third, the technical feasibility of deleting genes in the mesenchymal compartment of the uterus in an effort to understand decidualization and post-natal interactions with the overlying epithelium has been demonstrated. It is concluded that beta-catenin plays an integral role in selective P_4_-directed epithelial-mesenchymal communication in both the estrous cycling and gravid uterus.

## Background

The stromal/mesenchymal compartment of the endometrium performs a variety of tasks important for uterine physiology, including relaying specific aspects of steroid hormone signaling to the overlying epithelium. An example of such mesenchymal-to-epithelial signaling occurs in response to estradiol (E_2_) binding to and activating estrogen receptor (ESR1), inducing the expression of stromal-derived growth factors that stimulate epithelial cell cycle progression, hypertrophy, and initiating secretory functions (reviewed in [1]). In invasively implanting species, the stroma also undergoes decidualization during early pregnancy following embryo apposition and attachment to the uterine luminal epithelium, a process inherently regulated by progesterone (P_4_) following E_2_ priming. Here, stromal cells terminally differentiate and contribute to pregnancy by performing placenta-like functions until such time that the embryo develops its own nutrient and gas exchange apparatus, the placenta [2]. Stromal decidualization is regulated, in part, by cues derived from the epithelium such as Indian hedgehog.

It is thought that ESR1 mediates E_2_-initiated signaling in the uterus. However, it is generally understood that E_2_-initiated transcriptional and physiological changes occur in two phases [3]. The first occurs within 2–6 hours, and the second takes place between 24–72 hours. Although many E_2_-initiated transcriptional events require binding of ESR1 to estrogen response elements (ERE), many other genes are regulated in an ER-dependent, but ERE-independent fashion [4]. This suggests that ESR1 interacts with other transcriptional modulators that in turn interact with DNA to regulate gene expression at promoter sites distinct from EREs. Within the uterine epithelium, one such ESR1 interacting molecule is the transcriptional co-activator β-catenin [5,6]. The late transcriptional response to E_2_ is thought to be mediated, in part, by the ESR1:β-catenin interaction. Equally complex signaling mechanisms likely coordinate P_4_ responses, but such pathways are less clearly understood.

β-catenin is best known for its central role in the canonical wingless-type MMTV integration site family member (Wnt) signaling pathways and β-catenin is essential for development, transcription, cell adhesion and tumorigenesis [7]. In the absence of Wnt signaling, β-catenin is found in the cytoplasm either as a component that binds cadherins to α-catenin and the cytoskeleton at adherens junctions or in a complex with adenomatous polyposis coli (APC), axin, and glycogen synthase kinase 3β (GSK-3β), wherein it is phosphorylated and subject to ubiquitination and proteasomal degradation. Activation of frizzled receptors by Wnt ligands disrupts the APC complex and inhibits GSK-3β activity causing an accumulation of unphosphorylated (*i.e.*, activated) β-catenin, which promotes its nuclear localization and subsequent regulation of target gene expression [8]. β-catenin is therefore uniquely situated at a bottleneck in the Wnt signaling pathway.

Much of the focus of steroid hormone signaling studies in the uterus has been directed at the epithelial compartment. In the present study, the function of β-catenin in the stromal compartment was investigated in the contexts of steroid hormone action and stromal cell decidualization. Our findings reveal that conditional inactivation of β-catenin in endometrial stroma results in disrupted progesterone signaling and complete loss of stromal cell decidua-lization, indicating that steroid-dependent and β-catenin signaling pathways intersect to regulate postnatal uterine functions.

## Methods

### Animals

Animal protocols were approved by either the Massachusetts General Hospital or the Washington State University Institutional Care and Use Committee. For histology, mature (6–8 weeks old) ICR female mice were placed with intact ICR males of proven breeding capacity or with vasectomized ICR males. Female mice were considered day of pregnancy (DOP) or day of pseudopregnancy (DOPP) 0.5 upon observation of a vaginal seminal plug. Whole implantation sites (pregnancy) or mechanically decidualized uterine tissue (pseudopregnancy) were collected on days 4.5, 6.5 and 7.5 and prepared for RNA isolation or paraffin sectioning as described below. Decidualization was induced in pseudopregnant female mice by infusing 10 μl of sesame oil into the uterine lumen on DOPP 4.

The utility of the anti-Müllerian hormone type II receptor (*Amhr2*) promoter to drive Cre recombinase expression in mice during uterine decidualization was first established by crossing *Amhr2*^*Cre*^ transgenic mice (*Amhr2*^*tm3(cre)Bhr*/+^), kindly provided by Dr. Richard Behringer or purchased from the Mutant Mouse Regional Resource Center, with *Rosa-EYFP* reporter mice containing a yellow fluorescent protein gene downstream of a loxP-flanked stop sequence (*Gt(ROSA)26Sor*^*tm1(EYFP)Cos*^) [9]. To study β-catenin function in endometrial stromal tissue *Amhr2*^*Cre*^ mice were mated with mice harboring a *Ctnnb1* gene with exons 2–6 flanked by *loxP* sites (*Ctnnb1*^*tm2Kem/KnwJ*^ ; The Jackson Laboratories, Bar Harbor, ME; [10]). Double transgenic *Amhr2*^*Cre/+*^*;Ctnnb1*^*flox/+*^ offspring derived from this first mating were then crossed to generate conditional mutant (*Amhr2*^Cre/+^*;Ctnnb1*^*d/d*^) and control (*Amhr2*^*Cre/+*^*;Ctnnb1*^*d/+*^ or *Ctnnb1*^*flox/flox*^) littermates. Attempts were made to induce decidualization using both natural (pregnancy) and artificial (intrauterine sesame oil injections during pseudopregnancy; [11]) means in control mice expressing β-catenin in the stromal compartment, as well as in mutant *Amhr2*^*Cre/+*^*;Ctnnb1*^*d/d*^ mice. To study the proliferative/mitotic effects of steroids on endometrial tissue from control and conditionally mutant female mice, ovariectomies were performed between three and five weeks of age. One week later, female mice were treated subcutaneously with steroid hormones as indicated in the legend of each figure. Tissues were collected at specified times, prepared for paraffin sectioning and analyzed for proliferation (BrdU labeling) and/or mitosis (phospho-histone H3 expression) as described below.

### Messenger RNA and protein expression analyses

Total RNA was extracted from individual samples and reverse transcribed (Superscript II RT; Invitrogen, Carlsbad, CA) with oligo-dT primers following DNase I treatment to eliminate genomic DNA contamination. Standard RT-PCR was then performed for genes of interest using primer sets listed in Table 1*. β-actin* mRNA was used as an internal control and a mock RT was also included as a template to confirm the absence of genomic DNA contamination. Quantitative RT-PCR analysis of *Gli1, Ptch1, Ihh, Muc1*and *β-actin* mRNA levels, was performed using a Cepheid Smart Cycler II with primers specific for each gene (Table 1) and Q SYBR Green Supermix (Bio-Rad). Relative quantification of mRNA levels was determined in which ratios for each gene was established using *β-actin* as a reference gene.

**Table 1:** PCR primers

**Murine Gene**	**Primer sequence**	**Annealing temperature**	**Cycles**
*Pgr*	5’ATGGTCCTTGGAGGTCGTAA3’ 5’CACCATCAGGCTCATCC3’	60	34
*Esr1*	5’CCAAAGCCTCGGGAATG3’ 5CTTTCTCGTTACTGCTGG3’	56	33
*Esr2*	5’GGGCCTGTTCGCCAGACTGC3’ 5’CAGGGATTTTCTTGGC3’	56	33
*Ihh*	5’CGTGCATTGCTCTGTCAAGT3’ 5’CTCGATGACCTGGAAAGCTC3’	56	37
*Pct1*	5’CCTCCTTTACGGTGGACAAA 3’ 5’ GCCACATCAAGAGGTTTGGT 3’	56	40
*Gli1*	5’CCTGGTGGCTTTCATCAACT3’ 5’GTGGTACACAGGGCTGGAGT3’	56	38
*β-actin*	5’GATGACGATATCGCTGCGCTG3’ 5’GTACGACCAGAGGCATACAGG3’	60	26

For immunohistochemical and immunofluorescent analyses, whole implantation sites and mechanically decidualized uterine tissues were prepared from paraffin or frozen sections as previously described [11,12]. Frozen uterine tissue sections from *Rosa-EYFP* transgenic mice were counterstained with DAPI and viewed directly using fluorescence microscopy. Sections processed for immunofluorescence were incubated with anti-total β-catenin antibodies at a dilution of 1:200 (Abcam) followed by incubation with an Alexafluor 546 conjugated secondary antibody (Invitrogen) and mounting medium containing DAPI.

For immunohistochemical detection of the active form of β-catenin, phospho-histone H3, ESR1 and progesterone receptor (PGR), paraffin embedded uterine sections were prepared as described elsewhere [11]. Sections were incubated with primary antibody diluted [(1:100 for anti-active β-catenin (Millipore), 1:2000 for anti-phospho-histone H3 (Upstate Biotechnologies), 1:300 for anti-ESR1 (Santa Cruz) and 1:300 for anti-PGR (Dako)], then incubated with biotinylated secondary antibody (1:500; Santa Cruz Biotechnologies) and horseradish peroxidase-conjugated streptavidin (Vector Laboratories; Burlingame, CA). Sections were exposed to 3,3’-diaminobenzidine (DAB) substrate, counterstained with hematoxylin, dehydrated, and mounted for light microscopy. The mean ratio of mitotic epithelial cells in endometrial tissue sections was established by counting the number of phospho-histone H3-positive cells and dividing by the total number of cells (mean of three tissue sections obtained from three different regions of the uterus) following standard immunohistochemical detection. β-catenin expression was also assessed by immunofluorescence in primary human endometrial stromal cells (kindly provided by Dr. Bo Rueda, Massachusetts General Hospital) induced to undergo decidualization in vitro by provision of 100 μM cAMP, 36 nM 17β-estradiol benzoate, and 1 μM P_4_ for 12 days.

### BrdU labeling and analysis of cellular proliferation

To assess cell proliferation in the individual epithelial and stromal compartments, control and mutant female mice were first ovariectomized at three to five weeks of age and allowed to clear endogenous ovarian-derived sex steroids for at least one week. Female mice then received E_2_ (100 ng s.c. in 100 μl sesame oil) on two consecutive days to prime the uterus and six days later began one of the following steroid hormone treatments. For epithelial cell proliferation, female mice received a single s.c. injection of E_2_ (50 ng in 50 μl of sesame oil) (n = 5 controls, n = 3 mutants). For stromal cell proliferation, female mice were injected with P_4_ (1 mg s.c. in 100 μl sesame oil) for three consecutive days and the following day with E_2_ + P_4_ (50 ng and 1 mg respectively in 100 μl sesame oil)] (n = 8 controls and mutants). Then 16 hrs after the final steroid hormone injection, female mice were treated with 50 mg/kg body weight 5-bromo-2’-deoxyuridine (BrdU; i.p. in 250 μl saline) for 2 hours prior to euthanasia and uterine dissection. Tissues were prepared for paraffin embedding and one section (6 μm thick) from three different regions of each uterus were used to assess BrdU incorporation immunohistochemically using a BrdU staining kit (Invitrogen Corporation, Camarillo, CA) per manufacturer’s instructions. The mean percentages of BrdU positive cells in the luminal epithelium (LE) and stroma were calculated by establishing a ratio of BrdU positive cells divided by the total number of cells within the luminal epithelium or subluminal stromal compartment.

### Experimental replication and statistical analysis

Each experiment was independently replicated a minimum of three times with different mice in each experiment. Data in graphs represent the mean ± SEM from replicated experiments. Assignment of mice to each experiment was made randomly. Raw data were analyzed with GraphPad PRISM software (version 4.0) for simple comparisons. Mean values were considered significantly different when *p* < 0.05.

## Results

### β-catenin expression during uterine receptivity and stromal decidualization

**Figure 1:**
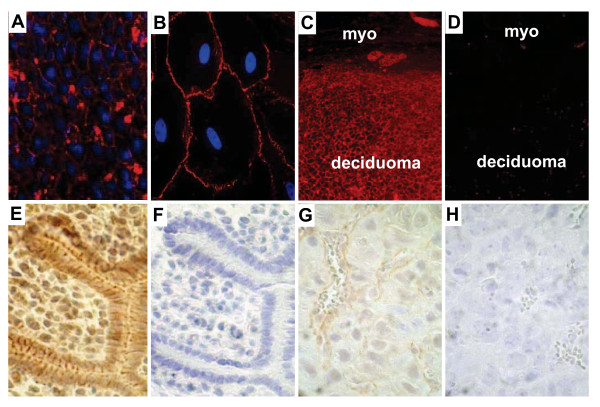
**Immunofluorescent detection of β-catenin during uterine decidualization.** Shown are representative images of total β-catenin immunofluorescent staining in transverse sections of implantation sites obtained on day of pregnancy (DOP) 7.5 (**A**), decidualized primary human endometrial stromal cells (**B**) and transverse sections of decidualized uterine tissue from day of pseudopregnancy (DOPP) 6.5 (**C, D**). Total β-catenin is expressed primarily at the cell surface of decidual stromal cells through the antimesometrial (**A**, 400X) and mesometrial (data not shown) poles on DOP 7.5, as well as in primary human endometrial stromal cells induced to undergo decidualization (**B**, 1000X). On DOPP 6.5 β-catenin is abundantly expressed in the stromal deciduum, but was below the level of detection in the myometrium (**C**, 200X). Immunohistochemistry of dephosphorylated (*i.e.*, active/nuclear) β-catenin was present on DOP 4.5 (**E**, 400X), but not DOP 7.5 (**G**, 400X). β-catenin was not detected in control sections stained without primary antibody (**D, F, H).** Representative micrographs from n = 3-5 independent experiments.

Our initial investigation showed that total β-catenin protein was observed throughout the implantation site on day of pregnancy (DOP) 7.5 (Figure 1A). The honeycomb expression pattern showed that β-catenin localized to the plasma membranes of decidualized stromal cells of both the antimesometrial and mesometrial compartments, consistent with its role in maintaining adherens function [13,14]. Similarly, expression of β-catenin was observed in primary human decidual cells (Figure 1B), as well as decidual cells of mechanically-induced pseudopregnant female mice on day of pseudopregnancy (DOPP) 6.5 (Figure 1C). We also analyzed whether localization of β-catenin to the nuclei of stromal cells changed with decidualization and observed that the active, dephosphorylated form of β-catenin was detected at the initial stages of decidualization on DOP 4.5 in both epithelial and stromal cells (Figure 1E). While the active form of β-catenin became localized to the membranes, cytoplasm and nuclei of epithelial cells on DOP 4.5, active β-catenin localized predominantly to the nuclei of stromal cells undergoing decidualization. This finding supports endocrine-dependent transcriptional and cell cycle regulatory roles for β-catenin in the stromal compartment as has been previously suggested based on the use of an ovariectomized rat model supplemented with steroid hormones [15]. By DOP 7.5, staining for active β-catenin was diminished in terminally differentiated decidual cells (Figure 1G). A similar expression profile was observed for active β-catenin on corresponding days of pseudopregnancy where decidualization was initiated by artificial means (data not shown). β-catenin was not detected in control sections where primary antibody was omitted (Figure 1D, F, H).

### Stromal β-catenin is necessary for establishing pregnancy

**Figure 2:**
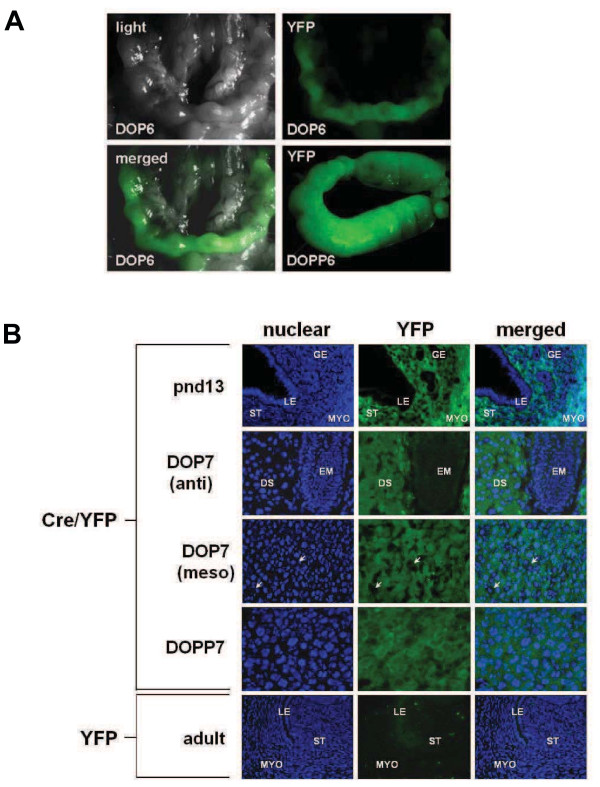
**Validating the use of the*Amhr2***^***Cre***^**transgenic mouse for studying gene function during uterine stromal cell decidualization.***Amhr2*^*Cre*^ transgenic mice were crossed with double floxed yellow fluorescent protein (Cre/EYFP) transgenic mice. (**A**) At sexual maturity female offspring were bred and implantation sites were observed grossly under fluorescent lighting on day of pregnancy 6 (DOP6, upper right and lower left panels) or day of pseudopregnancy 6 (DOPP6, lower right panel). (**B**) Direct fluorescent microscopy was used to assess frozen histological sections on postnatal day 13 (pnd13), DOP7 and DOPP7. Note that EYFP was present only in the stroma (ST) and myometrium (MYO) on pnd13 and DOP7 and absent in the luminal (LE) and glandular (GE) epithelia (pnd13). Likewise, EYFP was not detected in certain cells at the mesometrial pole that are presumably natural killer and endothelial cells (white arrows). EYFP was expressed at both the antimesometrial (anti) and mesometrial (meso) poles on DOP7. No EYFP was observed in *EYFP*^*flox/flox*^ single transgenic mice (EYFP) that lack cre recombinase expression (**B**, lower panels). All images taken at 400X magnification. DS, decidualized stroma; EM, embryo.

Because β-catenin expression increased dramatically in uterine stromal cells during decidualization, we hypothesized that this transcriptional co-activator functions to facilitate stromal cell differentiation. Complete β-catenin deficiency results in embryonic lethality [16]. Mice harboring the anti-Müllerian hormone type II receptor (*Amhr2*) gene promoter-driven *Cre recombinase* gene [17] were therefore used to conditionally delete *Ctnnb1* when mated with mice harboring floxed *Ctnnb1* alleles [10]. Stromal and decidual cell-specific activity of Cre recombinase was first confirmed using the floxed EYFP reporter mouse. As shown in Figure 2A, EYFP fluorescence was observed throughout the reproductive tract during early pregnancy (DOP 6), as well as in artificially stimulated deciduomal tissue of pseudopregnancy (DOPP 6) of double transgenic Cre recombinase expressing EYFP reporter mice (*Amhr2*^*Cre/+*^*;Rosa-EYFP*). As anticipated, EYFP was only observed in stromal cells of the endometrium from *Amhr2*^*Cre/+*^*;Rosa-EYFP* female mice on postnatal day 13, DOP 7 and DOPP 7, while vascular endothelial (eg., DOP 7 mesometrium) and epithelial cells were devoid of EYFP fluorescence (Figure 2B).

**Figure 3:**
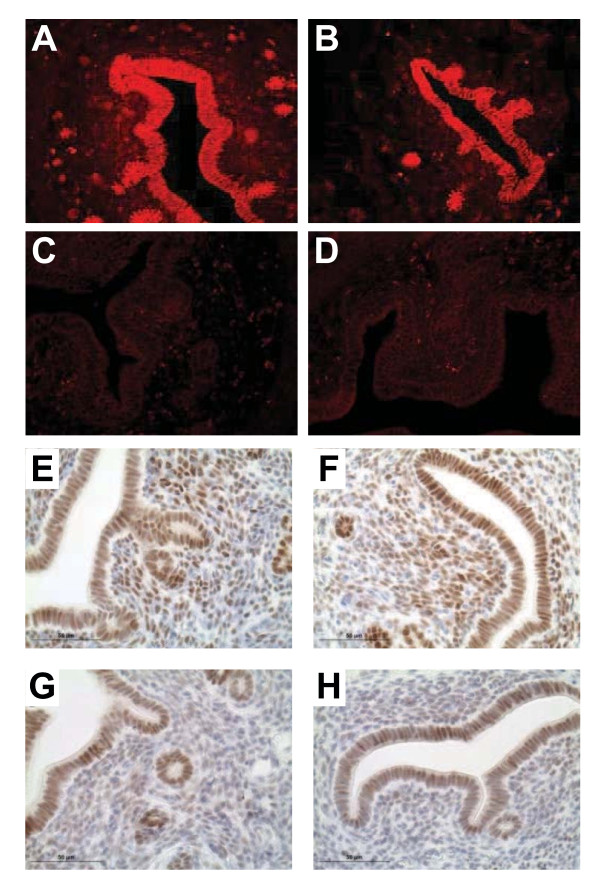
**Equitable steroid hormone receptor expression in control and mutant uteri.** Immunofluorescent detection of total β-catenin in *Ctnnb1*^*flox/flox*^ control (**A**) and *Amhr2*^*Cre/+*^*; Ctnnb1*^*d/d*^ mutant (**B**) uteri (200X). Total β-catenin was not detected in sections where primary antibody was omitted (**C, D**). Immunohistochemical detection of ESR1 in control (**E**) and mutant uteri (**F**), as well as PGR protein expression in control (**G**) and mutant (**H**) uteri. Representative micrographs from n = 3 independent experiments.

We next generated *Amhr2*^*Cre/+*^*;Ctnnb1*^*d/d*^ double transgenic conditionally null mice and used immunofluorescence microscopy to confirm restricted deletion of β-catenin from the stromal, but not epithelial compartment. Total β-catenin was observed in epithelia of both *Ctnnb1*^*flox/flox*^ control (Figure 3A) and *Amhr2*^*Cre/+*^*;Ctnnb1*^*d****/****d*^ mutant (Figure 3B) uteri, as well as in the sub-luminal stromal compartment of *Ctnnb1*^*flox/flox*^ uteri. Uterine tissues from control and mutant female mice maintained equitable potential to respond to female sex steroids in that they express similar levels of classical sex steroid hormone receptors based on immunohistochemical detection of ESR1 (Figure 3E, F) and PGR (Figure 3G, H), as well as mRNA expression of each receptor as determined by semi-quantitative RT-PCR (data not shown).

**Figure 4:**
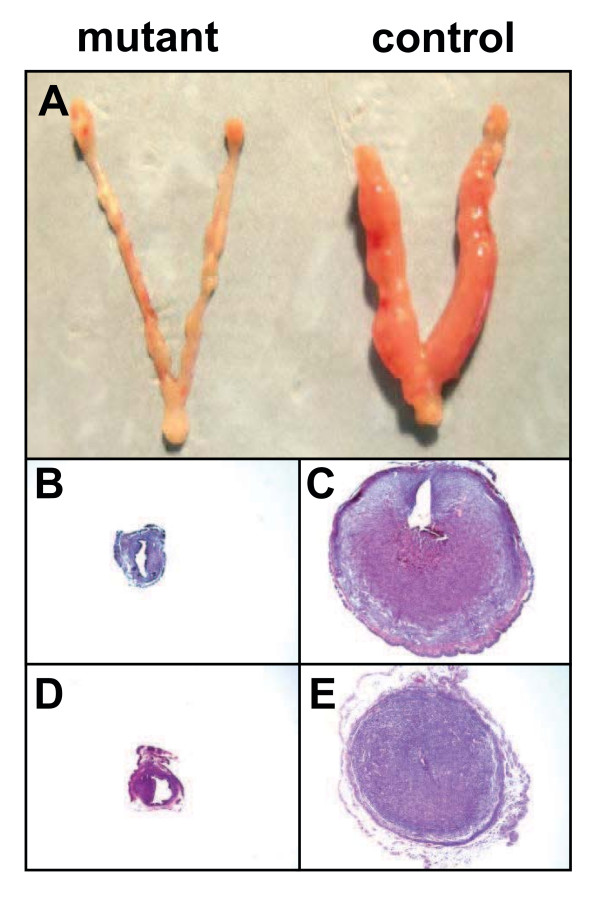
**Conditional deletion of*Ctnnb1*from the stromal compartment results in failed uterine decidualization.** (**A**) Shown are whole uteri isolated from control (*Ctnnb1*^*flox/flox*^) and mutant (*Amhr2*^*Cre/+*^*;Ctnnb1*^*d/d*^) female mice. (**B-E**) Histological transverse sections of uteri induced to undergo decidualization for 36 h. Uteri were obtained from intact control (**C, E**) or mutant (**B, D**) female mice in which endogenous steroids and pseudopregnancy primed uteri for the deciduogenic response (**B, C**), or from ovariectomized female mice receiving an exogenous hormone regimen consistent with that of early pregnancy [**D, E**; two days of E_2_ (100 ng s.c. in 100 μl sesame oil), two days no treatment, three days P_4_ (1 mg s.c. in 100 μl sesame oil) and one day E_2_ + P_4_]. Note that while uteri from control mice show clear signs of decidualization at 36 h post-induction (**C, E**), uteri from mutant mice do not decidualize (**B, D**). Representative results from n = 4-5 independent experiments.

*Amhr2*^*Cre/+*^*;Ctnnb1*^*d****/****d*^ female mice are infertile due in part to incomplete development of the oviduct [18,19]. To study the impact of β-catenin deficiency within the stromal compartment on uterine function, decidualization was induced artificially in female mice bred to vasectomized male mice on day of pseudopregnancy (DOPP) 4. This approach circumvented the need for an intact oviduct and embryo deposition into the uterus for the initiation of decidualization. While *Ctnnb1*^*flox/flox*^ control mice showed normal decidualization reaction 36–72 h after induction, uteri from *Amhr2*^*Cre/+*^*;Ctnnb1*^*d****/****d*^ female mice could not be stimulated to undergo decidualization (Figure 4A). Histological analysis confirmed this finding (Figure 4B-C). To rule out the possibility that uteri from *Amhr2*^*Cre/+*^*;Ctnnb1*^*d****/****d*^ mice fail to decidualize due to insufficient endogenous ovarian steroid hormone synthesis, ovariectomized *Ctnnb1*^*flox/flox*^ control and *Amhr2*^*Cre/+*^*;Ctnnb1*^*d****/****d*^ mice were exposed to exogenous steroids in a regimen that mimicked early pregnancy to induce artificial decidualization [20]. Steroid supplemented control mice show normal decidualization reactions at 36–72 h based on histological examination (Figure 4E), but uteri from *Amhr2*^*Cre/+*^*;Ctnnb1*^*d/d*^ mice were again incapable of undergoing decidualization (Figure 4D). These findings indicate that stromal β-catenin is essential for uterine decidualization.

### A role for stromal β-catenin in steroid hormone signaling

**Figure 5:**
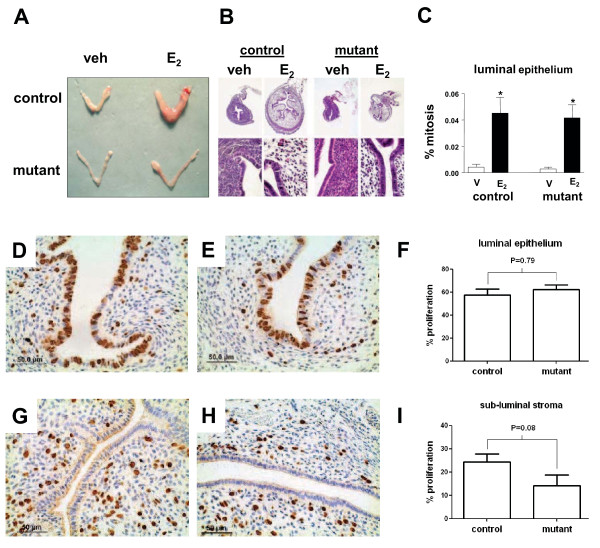
***Ctnnb1***^***flox/flox***^**control and*Amhr2***^***Cre/+***^***;Ctnnb1***^***d/d***^**mutant uteri responsd similarly to steroid treatments in terms of epithelial and stromal cell proliferation.** Representative images of whole uteri (**A**) or histological sections (**B**) from control or mutant female mice treated with vehicle (veh or v) or estradiol (E_2_, 100 ng, 24 h). (**C**) Based on immunohistochemical detection of the mitosis marker phospho-histone H3, no difference was observed in the percentage of epithelial mitotic cells between control and mutant mice exposed to vehicle or E_2_. When employing the BrdU labeling method, no statistical difference was observed in proliferation in control (**D, G**) and mutant (**E, H**) luminal epithelia in response to E_2_ (**D-F**; 50 ng, 24 h) or stromal cells in response to steroid hormones given in a regimen consistent with early pregnancy [**G-I**; two days of E_2_ (100 ng s.c. in 100 μl sesame oil), two days no treatment, three days P_4_ (1 mg s.c. in 100 μl sesame oil) and one day E_2_ + P_4_]). Data represent the mean ± SEM from n = 3-8 independent experiments where * denotes *p* < 0.05.

Decidualization requires exposure of the uterus first to E_2_ followed by P_4_. Since decidualization failed in *Amhr2*^*Cre/+*^*;Ctnnb1*^*d/d*^ mice, we next studied whether the loss of β-catenin interfered with steroid hormone signaling in control and mutant female mice. Gross (Figure 5A) and histological (Figure 5B) examination of uteri exposed to a single injection of E_2_ (100 ng, 24 h) revealed that uteri from both *Ctnnb1*^*flox/flox*^ control and *Amhr2*^*Cre/+*^*; Ctnnb1*^*d/d*^ mice become edematous, a normal physiological response to E_2_. A more subtle response to E_2_ was observed in *Amhr2*^*Cre/+*^*;Ctnnb1*^*d/d*^ uteri, but this likely stems from reduced early postnatal growth of uterine mesenchyme rather than from a lack of an E_2_ signaling cascade. This can be seen when comparing uteri from *Ctnnb1*^*flox/flox*^ and *Amhr2*^*Cre/+*^*;Ctnnb1*^*d/d*^ mice exposed to vehicle (Figure 5B) and is supported by an equal number of E_2_-induced epithelial cell mitoses (*i.e.*, phospho-histone H3 immunostaining) in control and mutant uteri (Figure 5C) suggesting normal E_2_-induced epithelial cell proliferation. This experiment was then repeated using the BrdU labeling (*i.e.*, marker of proliferation) approach and it was again determined that β-catenin-deficiency within the stromal compartment did not compromise epithelial cell proliferation (Figure 5D-F). Finally, control and mutant female mice were exposed to E_2_ and P_4_ in a regimen consistent with early pregnancy to determine if stromal cell proliferation was altered in the endometria of conditionally null *Ctnnb1* female mice. As shown in Figure 5G-I, stromal β-catenin-deficiency did not significantly reduce stromal cell proliferation in response to steroid hormones. Collectively, these results suggest that the lack of β-catenin in endometrial stroma does not materially affect steroid hormone-induced cellular proliferation or stromal imbibition.

**Figure 6:**
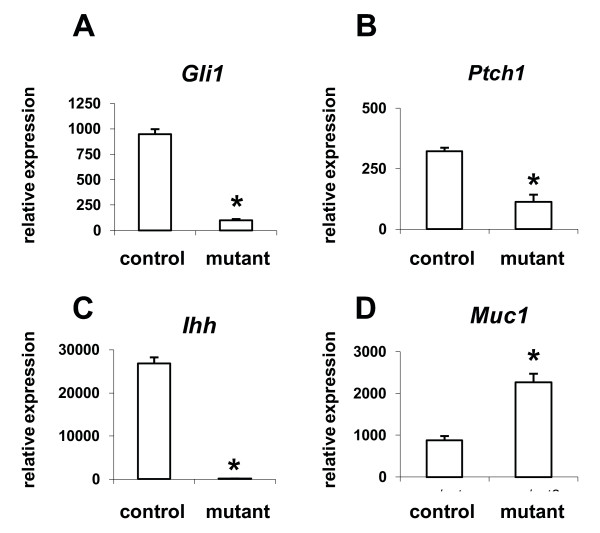
**Real time RT-PCR demonstrating differential mRNA expression of genes critical for embryo implantation.** Control and mutant uteri were obtained from female mice treated with a steroid hormone regimen consistent with early pregnancy [two days of E_2_ (100 ng s.c. in 100 μl sesame oil), two days no treatment, three days P_4_ (1 mg s.c. in 100 μl sesame oil) and one day E_2_ + P_4_]). Shown are real time RT-PCR results for *Gli1* (**A**), *Ptch1* (**B**), *Ihh* (**C**) and *Muc1* (**D**). Data represent mean + SEM of n = 4 independent experiments.

**Figure 7:**
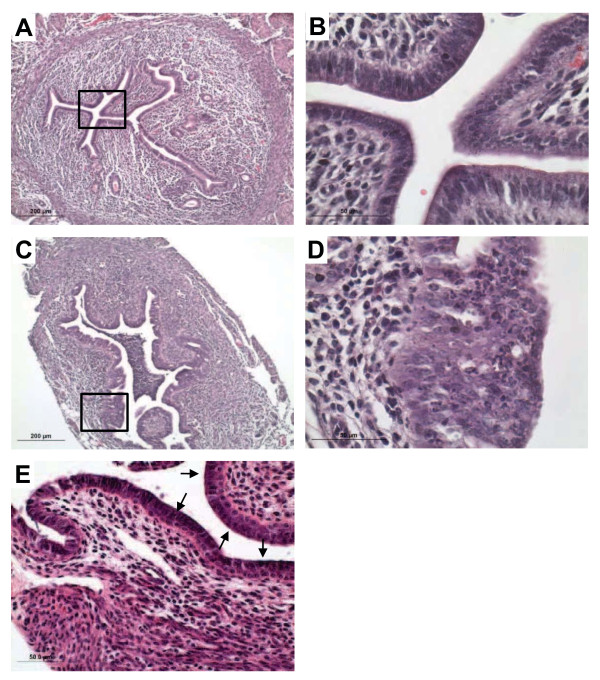
**Stromal β-catenin deficiency results in development of luminal metaplastic lesions in*Amhr2***^***Cre/+***^***;Ctnnb1***^***d/d***^**mutant uteri following steroid hormone treatment.** Hematoxylin and eosin stained sections from *Ctnnb1*^*flox/flox*^ control (**A, B**) and *Amhr2*^*Cre/+*^*;Ctnnb1*^*d/d*^ mutant (**C, D**) uteri following treatment of ovariectomized female mice with a steroid hormone regimen consistent with early pregnancy [two days of E_2_ (100 ng s.c. in 100 μl sesame oil), two days no treatment, three days P_4_ (1 mg s.c. in 100 μl sesame oil) and one day E_2_ + P_4_]. (**E**) Regions of bilaminar stratification (black arrows) were observed within the luminal epithelium in 66% (4/6) of the mutant uteri following E_2_ (100 ng, 24 h) treatment. Representative images from n = 6-9 independent experiments.

We next examined whether the failure of *Amhr2*^*Cre/+*^*;Ctnnb1*^*d/d*^ mice to undergo decidualization could be due to disruption of other critical signaling pathways that coordinate mesenchymal-to-epithelial communication. Mechanical decidualization was performed on 4–5 week old ovariectomized control and mutant mice for microarray analyses of P_4_-induced genes. Following treatment with a hormone regimen consistent with early pregnancy, the mRNAs of many known targets of P_4_ signaling during early pregnancy were disrupted in mutant uteri. One pathway in particular, the Indian hedgehog signaling pathway (*Gli1*, *Ptch1*, *Ihh*), was markedly reduced in mutant uteri (Figure 6A-C). In contrast, *Muc1*, a gene that is down-regulated at the time of embryo attachment and is a P_4_ target gene, remained elevated in mutant, but not control, uteri (Figure 6D). Interestingly, histological evaluation of transverse uterine sections 18 h after the final injection of combined E_2_ and P_4_ revealed that while control uteri showed the expected simple columnar epithelium and general tissue architecture (Figure 7A-B), uteri from 3/9 (33%) *Amhr2*^*Cre/+*^*;Ctnnb1*^*d/d*^ female mice displayed luminal epithelial metaplasia containing cystic structures and marked recruitment of leukocytes, particularly into the luminal space (Figure 7C-D). This observation prompted us to evaluate serial sections from control and mutant uteri exposed to E_2_ for 24 h. Here, regions of bilaminar stratification were observed within the luminal epithelium in 66% (4/6) of the mutant uteri (Figure 7E). Consistent with previous findings in which *Ctnnb1* was deleted using *Pgr-cre*[21], the posteriorization of the uterus shown here suggests that stromal β-catenin is necessary for maintenance of simple epithelial architecture within the uterus in response to E_2_.

## Discussion

Adult endometrial functions are temporally regulated by sex steroid hormones that require interplay between the epithelial and underlying stromal compartments. Ovarian-derived E_2_ generated during each estrous/menstrual cycle stimulates epithelial cell proliferation. The proliferative epithelial response to E_2_ is largely an indirect event that involves stromal release of epithelial mitogens such as IGF-1 [22,23]. It was recently established through conditional mutagenesis studies that stromal-derived ESR1 is fundamental for directing epithelial cell proliferation, while epithelial ESR1 is dispensable [24]. In turn, ovarian P_4_ completely abolishes E_2_-induced epithelial cell proliferation *in vivo*[25]. Clinically, P_4_ is applied prophylactically in some settings to treat estrogen-dependent endometrial cancer and to alleviate potential complications during hormone replacement therapies that can arise due to the unopposed actions of estrogens. These fundamental actions of E_2_ and P_4_ within the endometrium are further validated in pharmacological studies where steroid hormone actions are attenuated, as well as through the use of mutant mice deficient in expression of ESR1 and PGR.

*Amhr2*^*Cre/+*^*;Ctnnb1*^*d/d*^ mice are infertile, which engenders two previously unappreciated points for consideration. First, that deletion of *Ctnnb1* from the stromal, but not epithelial, compartment results in failed decidualization, suggests that this transcriptional co-activator mediates steroid hormone actions in the endometrium that are critical for fertility. Further investigation is needed to determine if β-catenin interacts in parallel with PGR, forming a complex that in turn regulates expression of genes in stromal tissue whose encoded products contribute to decidualization. Precedence for the convergence of β-catenin and steroid hormone signaling pathways has been established in the uterus. Alternatively, the PGR and β-catenin signaling pathways may work in series where PGR results in activation of another pathway, such as WNTs that in turn utilize β-catenin function. This scenario is supported by recent findings where WNT4 was shown to be a key regulator of normal postnatal uterine development and progesterone signaling during embryo implantation and decidualization [26]. Additional evidence for a PGR-β-catenin interaction comes from *in vitro* decidualization studies using human stromal cells where PGR expression was shown to be essential for nuclear translocation of β-catenin [27].

The second point for consideration is that stromal β-catenin is necessary for transcriptional regulation of both stromal and epithelial factors that are important for initiating decidualization and embryo attachment. Stromal β-catenin-deficiency results in failed up-regulation of *Ihh* in the epithelium, as well as *Ptch1* and *Gli1* in the stroma suggesting that stromal P_4_ signaling mediates events not only in the stromal compartment, but also in the overlying epithelium. It is concluded from this investigation that stromal β-catenin is a component of the signaling conduit through which P_4_ coordinates events in the overlying epithelium. Recent tissue recombination studies involving the use of wild type and *Pgr*-null stroma and/or epithelia support this concept [28]. Here, Simon *et al*. established that neonatal tissue recombinants containing wild type epithelium and PGR-deficient stroma were unable to show elevated levels if *Ihh* in the epithelium in response to P_4_ treatment [28].

Some studies have suggested direct inhibitory actions of P_4_ on E_2_-induced epithelial cell proliferation. During the time of embryo implantation on day 4 of pregnancy in mice the epithelium does not express PGR despite observation of clear progestational response on the epithelium [29,30]. How then does P_4_ signal in the epithelium in the absence of PGRs? The “progestamedin hypothesis” suggests that P_4_-induced paracrine factors secreted from the stromal compartment indirectly regulate P_4_ actions on the epithelium [30]. It was recently established that E_2_-induced epithelial proliferation is suppressed by P_4_ actions in the stromal compartment involving a HAND2-dependent mechanism [31]. Progesterone induces the transcriptional inhibitor HAND2, which in turn suppresses specific members of the fibroblast growth factors family in the stromal compartment [31]. Our study places β-catenin squarely in the middle of P_4_-dependent mesenchymal-to-epithelial signaling during the initiation of maternal:embryo interaction.

A number of signaling factors and down-stream transcription factors have been identified through mutant mouse studies as critical components coordinating decidualization. Some of these include IHH, WNT4, HOXA10, HOXA11, Src-kinase, BMP2 and COUP-TFII reviewed in [32]. Indian hedgehog localizes to the epithelium in response to P_4_ at the time of embryo implantation, and tissue restricted deletion of the gene using the *PgrR-Cre* mouse model results in failed decidualization [33-35]. From our study it is clear that transcription of members of the IHH pathway is reduced in *Amhr2*^*Cre/+*^*;Ctnnb1*^*d/d*^ uteri in response to steroid hormones; however, additional functional studies are necessary to determine exactly how β-catenin is linked to the IHH signaling pathway.

Uteri from *Amhr2*^*Cre/+*^*;Ctnnb1*^*d/d*^ mice are smaller in size than control uteri, which could confound the interpretation of these results. However, four lines of evidence suggest that the failure of *Amhr2*^*Cre/+*^*;Ctnnb1*^*d/d*^ uteri to decidualize stems from disruption of steroid hormone receptor signaling rather than from altered prenatal or early postnatal uterine development. First, expression studies reveal that uteri from *Amhr2*^*Cre/+*^*;Ctnnb1*^*d/d*^ mice have the potential to respond normally to E_2_ and P_4_ in that uterine mRNA and protein levels of ESR1 and PGR do not differ between control and mutant female mice. Second, uteri from control and mutant female mice display a normal response to E_2_, at least in terms of epithelial proliferation and stromal imbibition. Since the stromal compartment mediates the proliferative response in the epithelium, our findings indicate that the uterine stromal compartment of *Amhr2*^*Cre/+*^*;Ctnnb1*^*d/d*^ mice is fully capable of disseminating proliferative signals to the epithelium. Third, the actions of P_4_ are not completely ablated in the uteri of *Amhr2*^*Cre/+*^*;Ctnnb1*^*d/d*^ mice, since several genes previously shown to be targets of P_4_ action show the expected pattern of expression. For instance, *Hmga2* (high mobility group AT-hook 2), *Cdkl1* (cyclin-dependent kinase-like 1), and *Ldb2* (LIM domain binding 2) were shown to be down-regulated by P_4_ treatment *in vivo*[36]. Based on our microarray analysis each of these genes was down-regulated similarly in control *Ctnnb1*^*flox/flox*^ and mutant *Amhr2*^*Cre/+*^*;Ctnnb1*^*d/d*^ uteri *in vivo* (data not shown). Conversely, *S100a6* (calcyclin), *Irg-1* (immune responsive gene-1) and *Fst* (follistatin), three genes shown to be up-regulated by P_4_[37], were equitably up-regulated in *Ctnnb1*^*flox/flox*^ and *Amhr2*^*Cre/+*^*;Ctnnb1*^*d/d*^ uteri (data not shown). Fourth, indifferent stromal cell proliferation was observed in response to a hormone regimen consistent with early pregnancy. This suggests that the proliferative stromal cell response to P_4_ is not dependent upon β-catenin. In sum, these findings indicate that β-catenin deficiency in the stromal compartment of *Amhr2*^*Cre/+*^*;Ctnnb1*^*d/d*^ uteri results in aberrant gene expression of a specific cassette of P_4_-dependent genes, several of which belong to the IHH signaling cascade, but that other P_4_ responses are normal.

Two functional studies were previously published on β-catenin in the uterus. In the first, β-catenin activity was indirectly assessed through the use of *Tcf/Lef-LacZ* transgenic mice [38]. Here, β-galactosidase activity was used to identify coupling of β-catenin with the TCF/LEF transcriptional complex *in situ*. Based on this model, β-catenin activity was observed in the luminal epithelium and circular smooth muscle, an event that required the presence of an embryo. It was concluded that β-catenin was no longer active by late DOP5. However, β-catenin activity was defined by its ability to activate the *Tcf/Lef-LacZ* transgene, and β-catenin function was not addressed using deletional analysis (*e.g.*, gene knockdown or mutant mice deficient in β-catenin). Additionally, while we and others [14] have since demonstrated the presence of active (*i.e.*, dephosphorylated and nuclear) β-catenin in decidualizing stromal cells, Mohamed *et al*. were unable to detect transcriptional activity for the TCF/LEF complex in the stromal compartment, suggesting β-catenin may regulate gene expression within the stromal compartment by a TCF/LEF-independent mechanism.

More recently, Jeong *et al*. used the *Pgr-Cre* transgenic mouse line to delete β-catenin from PGR-expressing tissues, including all compartments of the uterus [21]. Using this model system, β-catenin deficiency in the entire uterus resulted in pleiotropic effects leading to infertility, most likely because of the inability of stromal cells to terminally differentiate and E_2_-induced morphological defects. The design, and therefore the conclusion, of our study differ to some extent from this previous report. First, while uteri from *Amhr2*^*Cre/+*^*;Ctnnb1*^*d/d*^ female mice lack expression of β-catenin in the myometrial and stromal compartments, as with the *Pgr-Cre* model, expression of β-catenin was retained in luminal and glandular epithelia using the *Amhr2*^*Cre*^ mouse line. Second, deletion of β-catenin in all compartments of the uterus resulted in metaplastic formation of the luminal epithelium in the intact mouse [21]. Analysis of the ovaries indicated that ovarian function was preserved. Jeong *et al*., concluded that β-catenin deficiency in the epithelium was the source of the metaplastic phenotype. This conclusion is well justified in that mutations in the human *Ctnnb1* gene are commonly associated with endometrial hyperplasia. Although our data does not rule out control of epithelial metaplasia by epithelial β-catenin, they indicate that, since *Amhr2*^*Cre/+*^*;Ctnnb1*^*d/d*^ mutant uteri also develop metaplasia, *albeit* with reduced severity and incidence, the lack of β-catenin in stroma alone can dictate formation of epithelial metaplasia. As with β-catenin, deletion of APC, a component of the β-catenin signaling pathway, from the uterine stromal compartment results in a more severe phenotype where endometrial hyperplasia and carcinogenesis are observed [39].

## Conclusion

Because β-catenin is connected to a multitude of cellular processes, we investigated the functional requirement of β-catenin in the stromal compartment of the endometrium for decidualization and responsiveness to steroid hormones. Our findings indicate that β-catenin is essential for early events in the terminal differentiation of uterine stromal cells. While it is well established that the stromal compartment indirectly coordinates epithelial cell proliferation through production of paracrine growth factors, deletion of stromal β-catenin did not alter E_2_-stimulated epithelial cell mitosis. Our study also provides evidence that the stromal compartment, through activation of β-catenin, mediates as least some of the actions of P_4_ on the epithelium. It will now be important to delineate upstream signaling pathways that activate stromal β-catenin and to identify β-catenin target genes that are necessary for disseminating steroid hormone actions on the epithelium, in addition to its role in decidualization within the stromal compartment.

## Competing interests

None of the authors have competing interests.

## Authors’ contributions

LZ completed most of the IHC and RT-PCR and some of the decidualization and histology experiments. AP completed experiments centered on progesterone and estrogen signaling, proliferative responses, receptor expression analyses, as well as some of the decidualization experiments. She also participated in drafting the manuscript. LZ assisted with IHC and animal husbandry. JT provided mice for these experiments and contributed to the experimental design and writing. JP was involved in all aspects of these studies and drafted the manuscript. All authors participated in editing and revising the manuscript. All authors read and approved the final manuscript.
